# SwissPalm: Protein Palmitoylation database

**DOI:** 10.12688/f1000research.6464.1

**Published:** 2015-07-16

**Authors:** Mathieu Blanc, Fabrice David, Laurence Abrami, Daniel Migliozzi, Florence Armand, Jérôme Bürgi, Françoise Gisou van der Goot

**Affiliations:** 1 Global Health Institute, School of Life Sciences, Ecole Polytechnique Fédérale de Lausanne (EPFL), Lausanne, CH-1015, Switzerland; 2 Bioinformatics and biostatistics Core Facility, School of Life Sciences, Ecole Polytechnique Fédérale de Lausanne (EPFL), Lausanne, CH-1015, Switzerland; 3 Proteomic Core Facility, School of Life Sciences, Ecole Polytechnique Fédérale de Lausanne (EPFL), Lausanne, CH-1015, Switzerland

**Keywords:** S-palmitoylation, palmitoyl-proteomes, database, proteomics, Acyl-biotin exchange, Acyl-RAC

## Abstract

Protein S-palmitoylation is a reversible post-translational modification that regulates many key biological processes, although the full extent and functions of protein S-palmitoylation remain largely unexplored. Recent developments of new chemical methods have allowed the establishment of palmitoyl-proteomes of a variety of cell lines and tissues from different species.  As the amount of information generated by these high-throughput studies is increasing, the field requires centralization and comparison of this information. Here we present SwissPalm ( http://swisspalm.epfl.ch), our open, comprehensive, manually curated resource to study protein S-palmitoylation. It currently encompasses more than 5000 S-palmitoylated protein hits from seven species, and contains more than 500 specific sites of S-palmitoylation. SwissPalm also provides curated information and filters that increase the confidence in true positive hits, and integrates predictions of S-palmitoylated cysteine scores, orthologs and isoform multiple alignments. Systems analysis of the palmitoyl-proteome screens indicate that 10% or more of the human proteome is susceptible to S-palmitoylation. Moreover, ontology and pathway analyses of the human palmitoyl-proteome reveal that key biological functions involve this reversible lipid modification. Comparative analysis finally shows a strong crosstalk between S-palmitoylation and other post-translational modifications. Through the compilation of data and continuous updates, SwissPalm will provide a powerful tool to unravel the global importance of protein S-palmitoylation.

## Introduction

S-palmitoylation is defined as the enzymatic attachment of a 16-carbon-chain palmitic acid to cysteine residues via a thioester bond ^1, 2^. S-palmitoylation increases the local hydrophobicity of proteins, driving their association with cellular membranes and their segregation to specific membrane domains ^3^ and also affect protein stability and protein-protein interactions ^4– 12^. S-palmitoylation appears to occur in all eukaryotes since it was found in yeast, parasites, worm, flies and plants. Importantly, it is the only reversible lipid post-translational modification (PTM) identified to date ^13^ and thus can dynamically regulate the function of proteins. For example, S-palmitoylation/S-depalmitoylation cycles control the shuttling of the small GTPases H-Ras and N-Ras between the Golgi and the plasma membrane thus regulating cell growth ^14, 15^. The dynamics of S-palmitoylation are mediated by the opposing activities of two families of enzymes: palmitoyltransferases (PATs), which catalyze the attachment of palmitate to specific cysteine residues while thioesterases detach it ^5, 16, 17^ and Acyl Protein Thioesterases (APTS) which remove the acyl chain.

Palmitoylated proteins are involved in key biological processes including ion transport, receptor function, membrane trafficking, signaling, cell growth, development, neuronal plasticity and immune response ^5, 18– 20^. Not surprisingly S-palmitoylation has been linked to a variety of human diseases, including neurological diseases such as Huntington’s disease ^21^, schizophrenia ^22^, Alzheimer’s disease ^23^ and cancer (gastric, bladder, lung, colorectal, carcinoma ^24– 29^). In addition, pathogens utilize host S-palmitoylation machinery to promote infection ^30^.

Despite the growing evidences of the importance of S-palmitoylation and the attractive therapeutic perspectives that it provides, the full extent of the S-palmitoylated proteomes is not well defined and our understanding of the mechanisms that regulates S-palmitoylation and its consequences on cellular and organismal levels function remains incomplete. This is due in part to several factors; technical difficulties in studying lipid modifications, the absence of a consensus sequence for S-palmitoylation identifiable with bioinformatics means and the lack of antibodies that would recognize S-palmitoylated cysteines, in analogy to phosphospecific antibodies. The field has therefore long relied almost exclusively on metabolic labeling with radioactive palmitate, immuno-precipitation of specific proteins, and autoradiography - sometimes requiring months of exposure times.

Novel methods now allow the capture of cellular or tissue palmitoyl proteomes followed by their identification by mass spectrometry. So far two major methods have been used. The first is Acyl Biotin Exchange (ABE), and its derivative Acyl Resin Assisted Capture (Acyl-RAC) ^31– 33^. It provides a snapshot of the S-palmitoylated proteins in a cell or tissue. It is based on the selective cleavage of thioester bonds, as found between the palmitate moiety and the cysteine residue, using neutral hydroxylamine (HA), and the subsequent capture of the liberated thiol groups with a thiol-specific reagent coupled - directly or indirectly - to beads. The second method is based on the metabolic labeling of S-palmitoylated proteins in live cells with palmitate analogues containing an azide or alkyne group, and thus will reveal the proteins that have undergone S-palmitoylation during the labeling time. After cell lysis, the labeled proteins can be isolated using click chemistry and affinity capture ^34^.

So far, 19 palmitoyl-proteomes have been published, identifying thousands of new putative S-palmitoylated proteins ^26, 31, 33– 49^, and many more are expected to be reported. Since these palmitoyl-proteomes were obtained from various cell lines and tissues originating from different species, the field is in need of integration of these datasets in order to compare them, identify orthologs, and analyze what conditions lead to changes in S-palmitoylation. Importantly, the above-described capture techniques not only differ in the population of S-palmitoylated proteins that they aim to capture but also in some drawbacks. For example, incomplete alkylation of proteins during ABE/Acyl-RAC isolation will lead to a poor enrichment ratio and thus false negatives, but this step is absent from the *in vivo* labeling followed by click chemistry protocol. Thus comparing studies performed with different techniques increases the confidence that a protein represents a true positive. Moreover, different studies have used different thresholds and criteria to establish their confidence levels. Finally, low-abundance proteins might be missed in studies in cells or tissues, while being enriched in studies on subcellular fractions.

To provide the scientific community with a tool to extract information from the comparison of different palmitoyl-proteomics studies, we have created SwissPalm, which combines results from large-scale palmitoyl-proteomics studies with curation from the literature of small S-palmitoylation studies. SwissPalm is a user-friendly web resource that allows users to search for proteins of interest through all published palmitoyl-proteomes, determine the predicted S-palmitoylation sites, identify orthologues, compare palmitoyl-proteomes and more. Combination of the available data raises the confidence that a protein of interest is indeed palmitoylated, as we have herein validated on the chaperone complex CCT. A palmitoylation database also provides the opportunity to compare with other databases, leading for example to the generalized cross-talk between palmitoylation and ubiquitination.

## Methods

### SwissPalm architecture

The database was designed to offer a general solution for storing knowledge on protein S-palmitoylation obtained from different types of studies: from biochemical studies focusing on a specific protein, to large-scale analyses by mass spectrometry-based proteomics.

***Protein hits and sites.*** In SwissPalm, two main objects have been designed to store the information related to S-palmitoylation. First, a *Hit* represents the knowledge that a given protein (or isoform) has been found as S-palmitoylated in a given study. Second, a *Site* is defined by a *Hit* and the position in the related sequence where the S-palmitoylation event was identified. A given *Hit* can have none, one or several associated sites. *Hit* and *Site* records are labeled with a unique identifier SPalmH# and SPalmS#, respectively. The enzyme(s) catalyzing the S-palmitoylation/S-depalmitoylation reactions on a *Hit* or *Site* can also be indicated.

***Protein sequences and annotations.*** Protein sequences are the primary and central source of information of the SwissPalm database. The UniProt Knowledge Base (UniProtKB) was chosen because it is a stable and regularly updated resource for protein sequences. One protein entry in UniProtKB can contain one or several sequences corresponding to alternative products (isoforms) of a single gene. Therefore, in the database, proteins and isoforms represent two distinct reference objects, and the known isoform-specific S-palmitoylation information can be reported to one or the other. The SwissPalm database is built for a set of species for which S-palmitoylation events have been reported for at least one protein. For each of these species, UniProtKB/SwissProt - the manually curated part of UniProtKB - sequences are inserted in the database. UniProtKB/TrEMBL entries are taken into account only if a palmitoyl-proteome hit protein is not found in UniProtKB/SwissProt. Moreover, UniProtKB provides curated functional annotations on proteins, *e.g.* ubiquitination or phosphorylation, which is useful to compare to S-palmitoylation. Information extracted from UniProtKB entries includes subcellular localization and sequence features (topological domains, variants, post-translational modifications, etc.). Post-translational modification information was also retrieved from Phosphosite ^50^.

Mappings to other protein databases like RefSeq or genome-specific databases ( *e.g.* The Arabidopsis Information Resource (TAIR), Mouse Genome Informatics (MGI), or *Saccharomyces* Genome Database (SGD)) were obtained either through the UniProt mapping API or from the databases themselves and inserted in SwissPalm as protein references. Gene Ontology (GO) terms and annotations were downloaded from the GO website and inserted in our database. A full mapping of UniProtKB entries and GO terms was computed, taking into account the lineage of GO terms and stored in the database. Thus, all UniProtKB entries associated with a given GO term can be found.

### Orthologies and computed data

Orthologs of *Hits* were extracted from OMA (Orthologs MAtrix) groups ^51^ and OrthoDB ^52^ and included in the database. Orthology relations are indicated in both databases at the level of proteins. For each protein, orthology groups represented in OrthoDB are listed non-redundantly. In OrthoDB, one protein can present several orthologs in the same species but in different orthology groups. To simplify the orthology information from OrthoDB, orthology groups were sorted by their size (number of orthologs in the group) from the smallest to the biggest, and only the first encountered protein for each species was selected. OMA group orthologs were used to complement this resource. These orthologs were used in the application for three purposes: the comparison of palmitoyl-proteomes of different species and the comparison of palmitoyl acyl transferases (PATs) from different organisms, and finally the analysis of conservation of known S-palmitoylation sites across different species.

Due to the increasing number of palmitoyl-proteome studies and of known involved proteins and orthologs, the comparison of palmitoyl-proteomes had to be precomputed to keep the website responding fast. The results of the comparison are stored in a specific table of the database, which is queried to present results in the palmitoyl-proteome comparison tool. For each protein, two types of multiple sequence alignments (MSA) were performed (when relevant) using MAFFT []: a MSA of isoform sequences and a MSA of protein orthologs (taking into account only the main isoform if several exist). For each sequence and each cysteine, a prediction of S-palmitoylation sites has been performed using two tools: PalmPred ^53^ and CSS-Palm 4.0 ^54^. Results are stored in the database.

### SwissPalm server and administrator interface

SwissPalm is a standard Ruby-on-Rails (RoR) application with a PostgreSQL database backend. The update procedure is fully automated and includes every step of download, parsing and loading in the database of the external and computed data. It is possible to initially update the list of species to load in the system. The application consists of a public website to browse and search the S-palmitoylation data in different contexts and an administration interface to manually add any curated information. The search is powered by a systematic indexation system of all known protein identifiers, all possible key-words from the UniProtKB description of proteins and GO terms. Mass spectrometry data from literature can be loaded through a specific function part of the SwissPalm server used in admin mode. The system uses the database to directly load the lacking sequences and validate the possibility of protein S-palmitoylation. The original file (tab-delimited format) is kept on the server file system, and in the database only internal protein/isoform IDs (corresponding to UniProtKB entries or sequences) are stored. A dedicated interface has been set up to allow the curation of hits and sites extracted from the literature. Several validators help to keep consistency in the database; for example, a systematic check is done at the level of the sequence to verify the presence of a cysteine at the indicated position.

### Bioinformatics analysis

***GO term, STRING, DAVID and CORUM analysis.*** GO terms, coming from RDAVIDWebService ^55, 56^, with enrichment scores >1.6 and corrected p-values <0.01 (using a Benjamini-Hochberg correction for multiple testing) were selected. In order to cluster GO terms by group of ontology, GO terms with more than ten proteins but fewer than 500 were selected. GO terms sharing the same biological functions enriched in S-palmitoylation Hit proteins were clustered using the R environment and the results were visualized with RCytoscape ^57^ and Cytoscape version 2.8.3 (Prefused forced direct layout) ^58^. The distance between GO terms corresponds to the inverse numbers of proteins common to the two terms. Uniprot IDs corresponding to the human and mouse palmitoyl proteomes were searched against the STRING database version 9.1 ^59^ for protein-protein interactions. Only interactions between the proteins belonging to the palmitoyl proteome dataset were selected using a confidence score ≥ 0.9 (high confidence). For CORUM analysis, complexes present in the CORUM database ^60^ with more than six proteins were selected and classified by the % of S-palmitoylation Hit proteins in these complexes.

***Two sample logo analysis.*** Sequence motif analyses were performed with the Two-Sample Logo software ^61^ using the 535 known sites of S-palmitoylation present in SwissPalm. Sequences collected in this study were compared with a set of 5000 same-length protein sequences containing cysteine residues randomly selected but not annotated or predicted as S-palmitoylated. Two groups of aligned sequences were statistically analyzed by the binomial test ( *p* < 0.01) against a binomial distribution.

### Experimental methods

In order to illustrate the potential of the SwissPalm database to increase the confidence level as to whether a given protein is palmitoylated or not, we have validated the palmitoylation of an identified protein complex, CCT. Similarly, the comparison of SwissPalm with ubiquitination databases suggested that proteins that can be palmitoylated also appear to be ubiquitinated. We therefore investigated this experimentally.

***Cells, Antibodies and Reagents.*** HeLa cells (human uterine cervical carcinoma) from ATCC were grown at 37°C in complete modified Eagle's medium (MEM) (Sigma), supplemented with 10% fetal bovine serum (FBS) (Brunschwig), L-glutamine, penicillin and streptomycin. HAP1 knockout cell lines were purchased from Horizon Genomics (Vienna, Austria) and were grown in complete Dulbecco’s MEM (DMEM) (Gibco), supplemented with 10% FBS, 2 mM L-glutamine, penicillin and streptomycin. The DHHC2 clone (09818-03) contains a 2bp insertion in exon 1, the DHHC5 clone (30129-12) contains a 10bp insertion in exon 2 and the DHHC6 clone (13474-01) contains a 5bp insertion in exon 2. Human Anti CCT1 (ab109126- Rabbit monoclonal, RRID:AB_10864216), CCT2 (ab92746- Rabbit monoclonal, RRID:AB_10565196), CCT3 (ab167559- Mouse polyclonal), CCT4 (ab49151- Rabbit polyclonal, RRID:AB_2073761), and CCT5 (ab129016- Rabbit monoclonal, RRID:AB_11154964) were obtained from Abcam. Human anti-α Ubiquitin (P4D1) (mouse monoclonal) was from Santacruz (sc-8017, RRID:AB_628423) and anti human α-actin (Mouse monoclonal) from Millipore (MAB 1501, RRID:AB_1675188).

***Immunoprecipitation.*** HeLa cells were lysed for 30 min at 4°C in IP buffer (0.5% NP-40, 500 mM Tris–HCl pH 7.4, 20 mM EDTA, 10 mM NaF, 2 mM benzamidine, and Roche protease inhibitor cocktail) followed by centrifugation for 3 min at 2000 *g*. The supernatants were pre-cleared with protein G-agarose-conjugated beads (GE Healthcare) and incubated for 16 h at 4°C with antibodies and beads. The beads were washed three times with the immunoprecipitation buffer and resuspended in sample buffer (2 ×) after the final wash. The samples were heated at 95°C for 5 min and migrated on SDS–PAGE. Western blotting was performed using the iBlot (Invitrogen) according to the manufacturer's instructions.

***Acyl-RAC.*** Protein S-palmitoylation was assessed by the Acyl-RAC assay as previously described ^33^, with some modifications. Briefly, total protein extracts (2 mg/ml) from cultured cells were incubated for 4 h at 42°C in buffer containing 1.25% SDS, 0.75% Triton X-100, Hepes 62.5 mM 1 mM EDTA, 20 mM methyl methanethiosulfonate (MMTS-Sigma), 8M Urea and protease inhibitor cocktail (Roche) at pH 7.4. MMTS was removed from the protein extract by chloroform–methanol precipitation followed by five methanol washes. Protein pellets were dried and solubilized in binding buffer (1% SDS, 1 mM EDTA, Hepes 100 mM, Urea 8M and protease inhibitor cocktail at PH 7.4). Supernatants were split into two: one sample was supplemented with 1 M hydroxylamine hydrochloride (pH 7.4) and 16.5 mg thiopropyl beads (Sigma); the other sample contained no hydroxylamine but 1M Tris (pH 7.4) and the same amount of thiopropyl beads. After overnight incubation at RT, beads were washed with washing buffer at least five times, and bound proteins were eluted by incubation of beads with 2 × SDS-PAGE loading buffer at 95°C for 5 min. Finally, samples were submitted to SDS-PAGE and analyzed by immunoblotting.

***Metabolic labeling with ^3^H-palmitic acid.*** HeLa cells were incubated for 2 h at 37°C in IM (Glasgow minimal essential medium buffered with 10 mM Hepes, pH 7.4) with 200 μCi/ml ^3^H-palmitic acid (9,10- ^3^H(N)) (American Radiolabeled Chemicals, Inc.). Cells were washed and the cell lysate was subjected to immunoprecipitation of the protein of interest as described above. After the washes, beads were incubated for 5 min at 95°C in reducing sample buffer (2 ×) prior to SDS–PAGE. After the SDS–PAGE, the gel was incubated with a fixative solution (25% isopropanol, 65% H _2_O, 10% acetic acid), followed by a 30 min incubation with signal enhancer Amplify NAMP100 (Amersham). The dried gels were exposed to a Hyperfilm MP (Amersham). Chemical removal of S-palmitoylation was performed by treating cell extracts for 10 minutes at room temperature with 1 M hydroxylamine hydrochloride (Sigma) pH 7.4.

## Results

### SwissPalm content

***Curated articles.*** By the time of submission, 303 published studies describing 365 S-palmitoylated proteins were incorporated into SwissPalm ( http://swisspalm.epfl.ch/hits). Strict criteria were applied to consider a protein as S-palmitoylated: proteins must have been demonstrated as S-palmitoylated by two independent methods or by one method and mutagenesis of S-palmitoylation sites. As a result, 535 known S-palmitoylation sites were included in SwissPalm. In addition, palmitoyltransferases and thioesterases demonstrated to be involved in the S-palmitoylation and S-depalmitoylation of those proteins were annotated.

**Figure 1.**
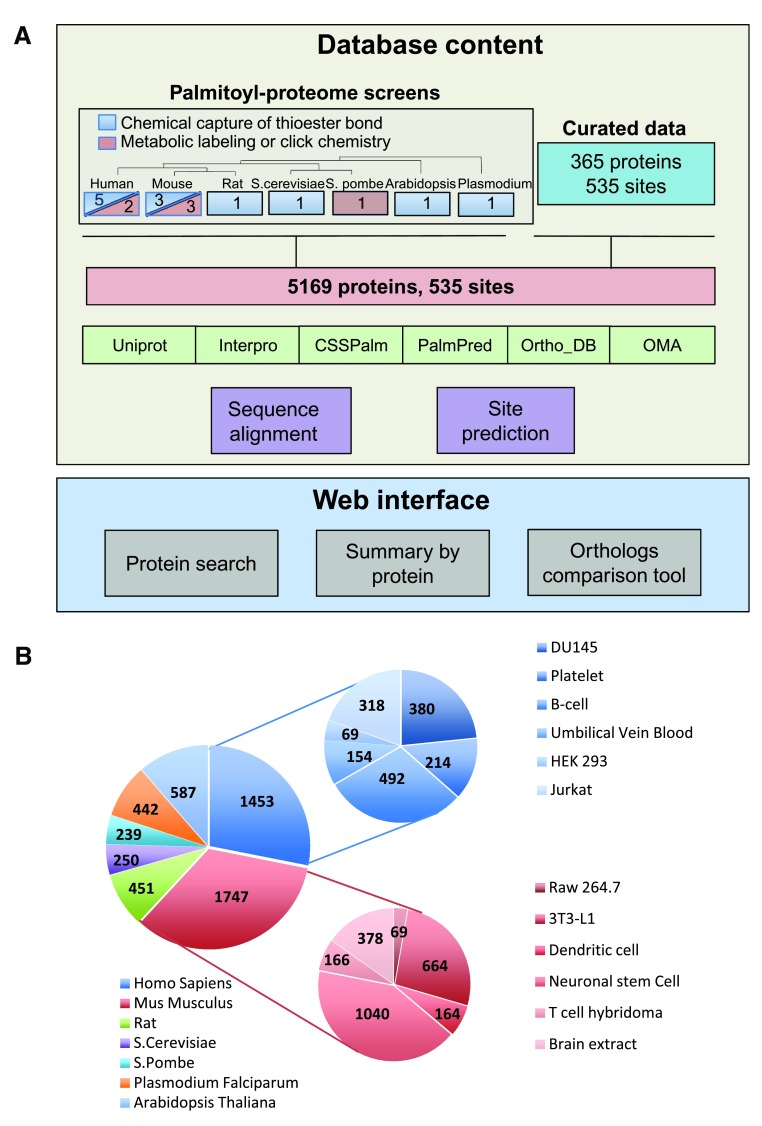
Palmitoyl-proteomes integrated in SwissPalm. **A**: Database content: Primary data on S-palmitoylation of proteins are extracted from MS large scale experiments on different species. Curated data on S-palmitoylation are obtained from the literature and input together with the MS information in the same data structure. In order to perform complex query related to S-palmitoylation, we have integrated in the database various external sources like orthology databases (Ortho_DB and OMA), UniProt features and subcellular localization information and Interpro domains. We ran also programs to have additional data, like multiple alignments of orthologous proteins or protein isoforms, and results from existing S-palmitoylation site predictors (CSS-Palm 4.0 and PalmPred). Web interface: The web interface presents the knowledge on S-palmitoylation in protein-centric pages. These pages are accessible through queries on a search engine. Some tools to analyse S-palmitoylation datasets are available online, like the orthologs comparison tool, aiming to perform cross-species S-palmitoylation comparison. **B**: 19 palmitoyl-proteomes from 7 species and various cell types and tissues were selected from published literature and integrated to SwissPalm. In total the dataset includes 5199 proteins.

***Palmitoyl-proteomes.*** 19 palmitoyl-proteome screens using ABE-, Acyl-RAC- or click chemistry-based studies were selected from published literature (for details and references see: http://swisspalm.epfl.ch/studies?large_scale=1). They cover seven species and ten palmitoyl proteomes performed with ABE, 2 with Acyl-RAC and 7 with click chemistry ( Figure 1A).

In all cases, the specificity of the capture is controlled by the addition of hydroxylamine (HA). The enrichment ratio (+HA/-HA) for ABE and Acyl-RAC and (-HA/+HA) for click chemistry samples determines the likelihood of proteins to be S-palmitoylated and the cut off in these high-throughput datasets is defined arbitrarily. Accordingly to the vast majority of the palmitoyl-proteomes studies, a ratio (+HA/-HA) greater than two for either ABE, and Acyl-RAC, or a ratio (-HA/+HA) greater than two for click chemistry was selected to add proteins into SwissPalm. In some studies, authors have classified their dataset of S-palmitoylated proteins into high confidence (HC, (+HA/-HA ratio > 20), Medium confidence (MC, 5 < ratio < 20) and low confidence (LC, 2 < ratio < 5) groups. The same classification was kept and annotated in SwissPalm, according to each study. As a result, 5199 unique proteins were incorporated into SwissPalm, all of them showing a ratio +HA/-HA higher than two in each case. The human dataset contains 1453 annotated hits from six different cell types (DUI145, Platelet, B-cell, Umbilical vein blood, HEK 293, Jurkat) while the mouse dataset comprises 1747 proteins from six different cell types or tissues (RAW 264, 3T3-L1, Dendritic cell, Neuronal stem cell, T cell hybridoma, Brain extract) ( Figure 1B).

**Figure 2.**
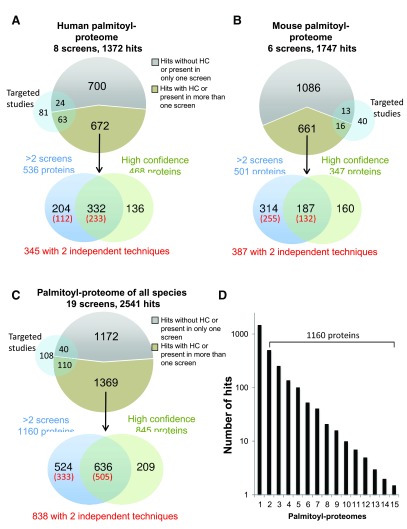
Improved confidence in S-palmitoylation protein hits. **A**: Analysis of the 1372 human hits contained in 8 palmitoyl proteomes: 672 S-palmitoylation hits are present in at least 2 human palmitoyl proteomes or are annotated as “high confident hits” (HC). 204 are only found in at least 2 human palmitoyl proteomes, 136 are only classified as HC and 332 S-palmitoylation hits are both found in more than one palmitoyl-proteome and classified as HC. Out of the 672 hits, 345 are identified with 2 independent techniques. 63 out of the 672 S-palmitoylation hits have been validated in targeted studies, while 24 out of 700 hits only found in 1 human palmitoyl proteome have been validated. **B**: Analysis of the 1747 mouse hits contained in 6 palmitoyl proteomes as described in **A**. **C**: Analysis of the 2541 human orthologous hits contained in 19 palmitoyl proteomes as described in **A**. **D**: Number of the S-palmitoylation hits by the occurrence of palmitoyl-proteomes in which they have been identified.

***SwissPalm-mediated improvement of confidence.*** The information generated by independent palmitoyl-proteome studies was used to build a filter system that increases the hit confidence. First we annotated the proteins that are defined as high confidence by the authors of the studies (468 for human and 347 for mouse) ( Figure 2A and Figure 2B, Supplementary table S1A and Supplementary table S1B). Second, we assume that the likelihood of a protein to be a true positive increases with its presence in multiple independent palmitoyl-proteomes. It is however important to keep in mind that even if proteins were isolated using the ABE/Acyl-RAC method of labeling followed by click chemistry, all proteins that contain a thioester bond, not related to S-palmitoylation, will be recovered and thus constitute false positives.

536 human proteins and 501 mouse proteins were found in at least two independent palmitoyl-proteomes ( Figure 2A and Figure 2B, Supplementary table S1A and Supplementary table S1B). To exploit the information from palmitoyl-proteomes contained in other species, we looked for human orthologs of S-palmitoylated proteins from other species. Out of the orthologs of 2541 human hits in SwissPalm, 1160 were present in at least two independent palmitoyl proteomes ( Figure 2C, Supplementary table S2). Well-studied S-palmitoylated proteins such as calnexin (CALX), Thioredoxin-related transmembrane protein 1 (TMX1), Synaptosomal-associated protein 23 (SNAP23), Ras-related protein (RRAS), Phosphatidylinositol 4-kinase IIα (P4K2A), transferrin receptor (TFR1), G-proteins (GNAI2, GNAI3), Ras related proteins were identified in multiple palmitoyl-proteome screens (at least in 10 out of 19) in at least four different species ( Table 1, Supplementary table S3) ^6, 50, 62– 67^.

**Table 1.** Top 20 proteins present in the palmitoyl proteome across the seven species.

UniProt ID	# of palmitoyl- proteome studies/HC hits
**CALX_HUMAN**	**15 / 7**
**SCAM3_HUMAN**	**14 / 7**
**GNAI2_HUMAN**	**14 / 6**
**TMX1_HUMAN**	**13 / 7**
**SNP23_HUMAN**	**13 / 6**
**LYRIC_HUMAN**	**13 / 6**
**RAP2B_HUMAN**	**12 / 5**
**RAP2C_HUMAN**	**12 / 6**
**P4K2A_HUMAN**	**12 / 4**
**GNAI3_HUMAN**	**12 / 5**
**GNA13_HUMAN**	**12 / 5**
**RRAS_HUMAN**	**11 / 4**
**TFR1_HUMAN**	**11 / 3**
**STX12_HUMAN**	**11 / 6**
**GNAQ_HUMAN**	**11 / 5**
**VAMP3_HUMAN**	**11 / 4**
**ERGI3_HUMAN**	**11 / 5**
**CYB5B_HUMAN**	**11 / 4**
**RAP2A_HUMAN**	**10 / 5**
**RASN_HUMAN**	**10 / 3**

Strikingly, ≈ 90% (1255 proteins) of the proteins present in more than one screen or annotated as high confidence hits have not yet been validated in targeted studies. In addition, more than 40% (524 proteins) of the proteins present in more than one screen were not annotated as high confidence hits in any of the studies ( Figure 2D, Supplementary table S2). Finally, we annotated the proteins that were identified by two distinct independent techniques (metabolic labelling and ABE-based methods), the overlap of false positive proteins generated by these two techniques being very limited. This corresponded to 345 proteins for human and 387 proteins for mouse ( Figure 2A and Figure 2B; Supplementary table S1A and Supplementary table S1B).

Altogether, using multiple screens, independent methods and extending the Hits by an ortholog search, we have identified a list of high confidence S-palmitoylated protein hits, which could be useful for further study.

### SwissPalm website

**Figure 3.**
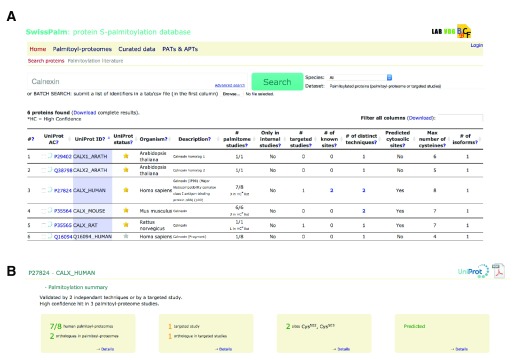
Search and result page. **A**: SwissPalm search page: Example of query for “calnexin” shows that it has been found in palmitoyl-proteomes from several species: human (7 out of 8 screens), mouse (6 out of 6), rat (1 out of 1) and *Arabidopsis thaliana* (1 out of 1). For human, mouse and rat calnexin was classified as a high confident hit and for human and mouse identified by two independent techniques (metabolic labeling and chemical capture). Finally, calnexin S-palmitoylation was also subject to targeted studies and 2 cysteine residues (502 and 503 in human calnexin) were identified. **B**: Results Page from human calnexin display summary boxes containing the main information related to S-palmitoylation: number of occurrences in palmitoyl-proteome screens and targeted studies, sites information, cysteine prediction.

***Search page.*** The web-based search tool is accessible from any page of the SwissPalm website: www.swisspalm.epfl.ch and produces as output a list of proteins with a direct link to detailed information for each protein ( Figure 3A). Protein searches are initiated by submitting a protein name, gene name or Uniprot_ID in the query section and protein IDs that match the string search will be returned in the results sections. Protein searches can be restricted to specific criteria. These include: ‘species’, ‘presence in palmitoyl-proteome screens’, ‘predicted to be S-palmitoylated’ or ‘obtained from a third party application/tool’ ( *e.g.* from a complex query on the UniProt website). Search can also be restricted to the “reviewed” annotated UniProtKB/SwissProt proteins. Batch searches are possible by submitting a list of identifiers in a tab/csv file (in the first column). An advanced search tool is available to help the user choosing among identifiers recognized by the search engine. Also, it presents controlled vocabularies that can be used to perform complex queries within the database (Motif Search, GO term and Subcellular localization).

**Figure 4.**
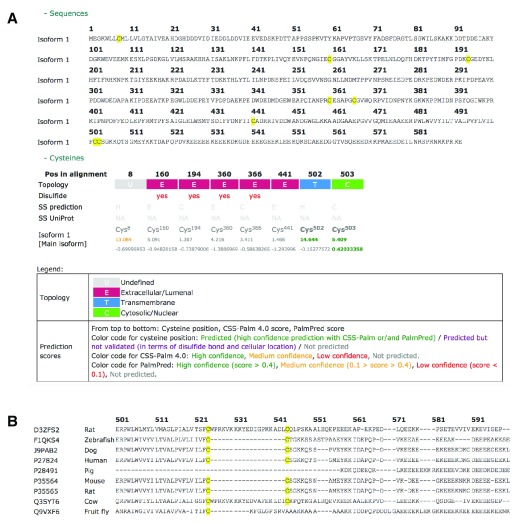
Additional information. **A**: (upper) Global alignment of isoform sequences highlighting all cysteine residues. (lower) When available, information on protein topology, disulfide bond involvement and prediction scores from CSS-Palm 4.0 and PalmPred are provided for each cysteine residue in the different isoform sequences. **B**: Global alignment of orthologs sequences show conserved cysteine residues (502 and 503 in human calnexin) across species.

***Protein information page.*** A system of summary boxes gives a quick overview on the main information related to S-palmitoylated proteins. This includes the number of times the protein is cited in proteome or targeted studies, information on experimental sites, and high confidence predictions from CSS-Palm 4.0 and PalmPred ( Figure 3B). Other information includes a global alignment of isoform sequences highlighting all cysteine residues ( Figure 4A), protein topology, disulfide bond positions and prediction scores ( Figure 4B), information on orthologous proteins and an alignment of them ( Figure 3D), as well as GO terms and references (cell types, techniques, subcellular localization).

### Data set analysis

**Figure 5.**
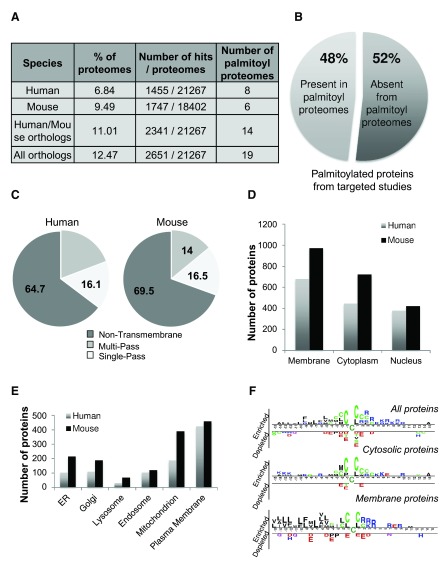
Abundance and distribution of S-palmitoylated proteins in the mammalian proteome. **A**: Percentage of S-palmitoylation hits combined from 8 human or 6 palmitoyl proteomes in the human and mouse proteomes. The analysis was extended to human orthologs of mouse and all S-palmitoylation hits present in the dataset. **B**: Percentage of targeted studies present in palmitoyl-proteomes. **C**: Topology of human and mouse S-palmitoylation hits. **D** and **E**: Distribution of human and mouse S-palmitoylation hits in cellular compartments. **F**: Enrichment of amino acid nearby validated S-palmitoylated cysteines in all, only cytoplasmic or only membrane proteins.

***Estimation of the human palmitome.*** We made use of the information gathered in SwissPalm to obtain a current estimation of the human palmitoyl-proteome. We found using Uniprot annotation that 6.8% of the human (1453 proteins) and 9.5% (1747) of the mouse proteomes may undergo S-palmitoylation ( Figure 5A, Supplementary table S3). Since several identified S-palmitoylation sites are conserved across species, we integrated 891 mouse orthologs that were not present in human palmitoyl proteomes. The joint dataset indicates that 11% (2339 proteins) of the human proteome may undergo S-palmitoylation. Extending the analysis to 2649 human orthologs present across all species, 12.47% of the human proteome may be subject to S-palmitoylation ( Figure 5A, Supplementary table S3). Moreover, since more than half of the proteins described in published targeted studies were not identified in any of the palmitoyl proteome screens ( Figure 5B), the current figure of 9 to 12% is probably an underestimate.

**Supplementary figure S1.**
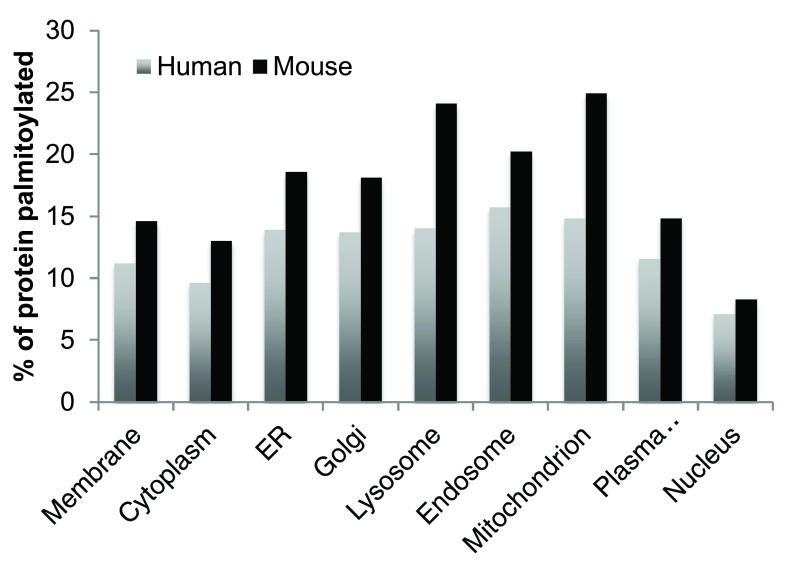
Percentage of human and mouse S-palmitoylation hits in cellular compartments.

***Protein localization and S-palmitoylation motif.*** Using the UniProt annotation on protein localization, 45% of both the human and mouse S-palmitoylation hits (679 and 974 for proteins respectively) were annotated as membrane proteins, 30% as cytosolic (445 and 723 proteins respectively) ( Figure 5D). This represents a modest enrichment in membrane proteins, more specifically of Type I membrane proteins, when compared to the total human and mouse proteomes ( Figure 5C, Supplementary table S4, Supplementary figure S1). S-palmitoylation hits are found in all major compartments of the endomembrane system – endoplasmic reticulum, Golgi apparatus, lysosome, endosome – as well as in mitochondria ( Figure 5E). The latter finding is intriguing since none of the DHHC palmitoyl transferases have so far been localized to mitochondria ^68^.

Additionally, 377/1453 human and 422/1747 mouse proteins identified in palmitoyl proteomes were associated with the nucleus which represents around 25% of the total S-palmitoylated human and mouse proteins ( Figure 5D). While these might be cytosolic proteins associated with the outer nuclear membrane, the higher number of nuclear proteins raises the possibility that S-palmitoylation may occur in the nucleus and could be used to reversibly target nuclear proteins to the inner nuclear membrane. Presence of a palmitoyltransferase in the nuclear envelope has been reported for the yeast protein rif1 ^69^. Palmitoylation of nuclear proteins will clearly require validation.

In order to investigate whether certain amino acids were preferentially enriched around S-palmitoylation sites, we compared the amino acid environment of the 535 sites present in SwissPalm against a random set of cysteines not predicted as S-palmitoylated (Materials and methods). A two sample logo analysis shows that S-palmitoylated cysteines are preferentially surrounded by other cysteines, have hydrophobic amino acid to the N-terminal side and charged amino acid to C-terminal side of the S-palmitoylated cysteine ( Figure 5F). We repeated the analysis, separating soluble from transmembrane proteins. With the exception of neighboring cysteines, no amino acid specificity was found for soluble proteins. For membrane proteins, despite the fact that we did not classify them as type I, II or multispanning, S-palmitoylation sites appear to preferentially locate to the C-termini of transmembrane domains, there appears to be multiple cysteines and these are preferentially followed with charged residues. This residues could contribute to the inside positive rule of membrane proteins and also ensure interaction with the cytosolic domain with negatively charged lipid head groups at the plasma membrane for example, as observed for myristoylated proteins ^70^.

This analysis does not exclude the presence of specific motifs that would ensure S-palmitoylation by a given palmitoyltransferase. Such an analysis will require extensive knowledge of the specific target of the DHHC enzymes.

Altogether this analysis indicates that S-palmitoylation is not restricted to a specific compartment and that it is slightly more frequent in membrane proteins.

**Supplementary figure S2.**
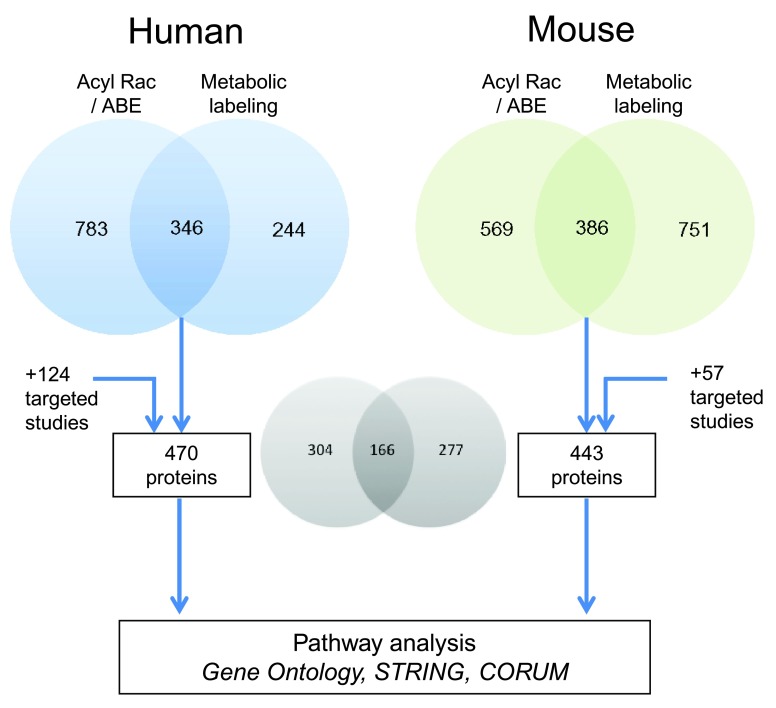
Strategy to obtain a high confidence list of 470 human and 443 mouse S-palmitoylation hits to perform ontology and network analysis of S-palmitoylation hits.

***Ontology and network analysis.*** To search for cellular functions enriched in S-palmitoylated proteins, we performed pathway- and network-based analysis on both human and mouse palmitoyl-proteomes. Gene ontology ^56, 71^ and protein-protein interaction analysis (through the STRING database) were performed on proteins found by two independent techniques or by targeted studies: 470 proteins for human and 443 for mouse ( Supplementary figure S2).

**Figure 6.**
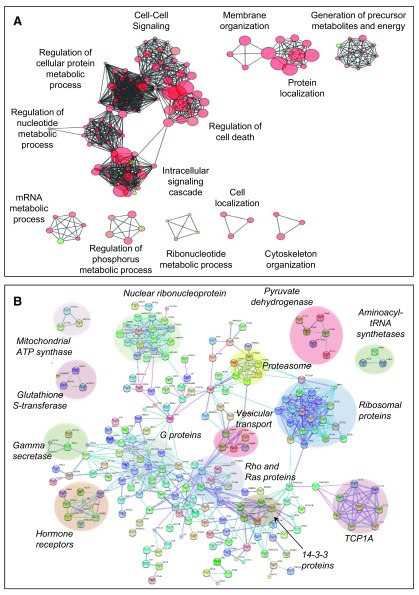
Ontology and network analysis of S-palmitoylated proteins. **A**: GO term analysis of 470 human S-palmitoylation hits found by 2 independent techniques or by targeted studies. GO terms sharing the same biological functions enriched in S-palmitoylation Hit proteins are clustered. The distance between GO terms corresponds to the inverse numbers of proteins common to the two terms and the size of the circle to the number of proteins associated with the GO term. **B**: Protein-protein interactions networks analysis of 470 human S-palmitoylation hits found by 2 independent techniques or by targeted studies using STRING software. The interactions (high confidence score > 0.9) are shown in evidence view (pink: experimental evidences and blue database evidences).

**Supplementary figure S3.**
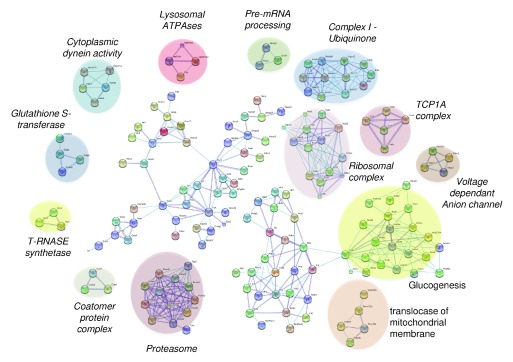
GO term analysis of mouse palmitoyl-proteome hits. GO term analysis of 443 mouse S-palmitoylation hits found by 2 independent techniques or by targeted studies. GO terms sharing the same biological functions enriched in S-palmitoylation Hit proteins are clustered. The distance between GO terms corresponds to the inverse numbers of proteins common to the two terms and the size of the circle to the number of proteins associated with the GO term.

**Supplementary figure S4.**
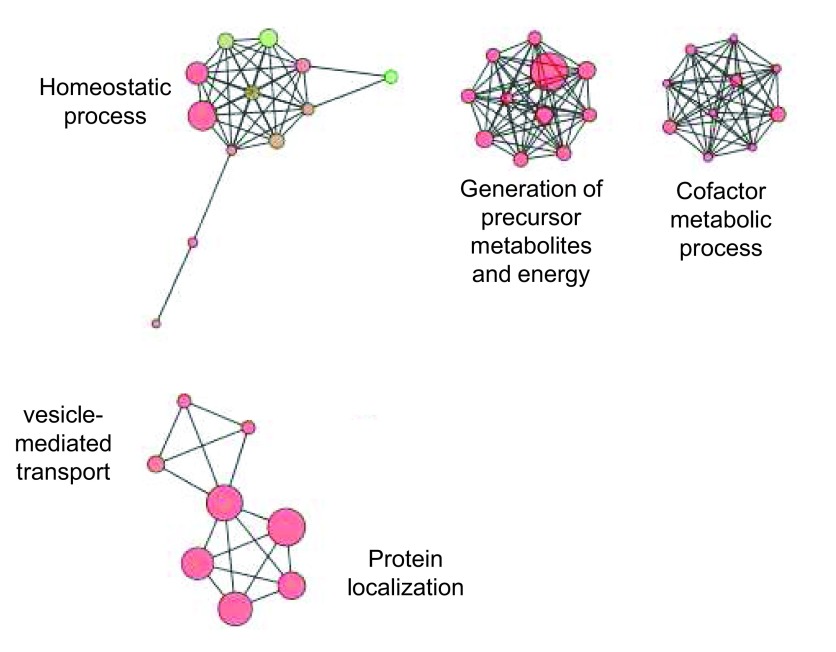
Protein-protein interaction networks within mouse S-palmitoylated proteins. Protein-protein interactions networks analysis of 443 human S-palmitoylation hits found by 2 independent techniques or by targeted studies using STRING software. The interactions (high confidence score > 0.9) are shown in evidence view (pink: experimental evidences and blue database evidences).

Consistent with the findings reported in some targeted studies, specific biological functions/processes ( Figure 6A, Supplementary figure S3 and Supplementary figure S4, Supplementary table S6, methods) are enriched in potentially S-palmitoylated proteins: membrane organisation, cytoskeleton organization, protein localization, cell localization and cell signalling. For example, proteins involved in vesicular trafficking were enriched such as subunits of the coatomer complex, clathrin as well as a significant number of SNARE proteins ( Supplementary table S5). We also found 289 hit proteins associated with the GO term Cytoskeleton. These include microfilament- and microtubule-related proteins such as actin subunits, dynein and vimentin but also proteins involved in the formation and regulation of these structures: profilin I, Arp2-Arp3 related proteins. Consistent with a role of S-palmitoylated proteins in cytoskeleton dynamics, tubulin S-palmitoylation was shown to contribute to its association with membrane ^72^ ( Supplementary table S5). More recently, S-palmitoylation of LIM Kinase-1 was shown to regulate spine-specific actin polymerization and thereby morphological plasticity ^73^. The full view of the role of S-palmitoylation in cytoskeleton organization will clearly require further studies.

More unexpected processes were also found to be enriched: regulation of cell death, metabolism, mRNA metabolic processes, generation of precursor metabolites and energy, ribonucleotides metabolic processes ( Figure 6B, Supplementary table S5 and Supplementary table S6). Whether this is due to the abundance of these proteins or a functional requirement for S-palmitoylation remains to be investigated. It is tempting to speculate that S-palmitoylation might regulate the association of large cytosolic protein complexes, such as ribosomes or proteasomes with membranes to ensure an efficient regulation of protein synthesis and degradation ( Figure 6B, Supplementary table S5) ^74^.

**Figure 7.**
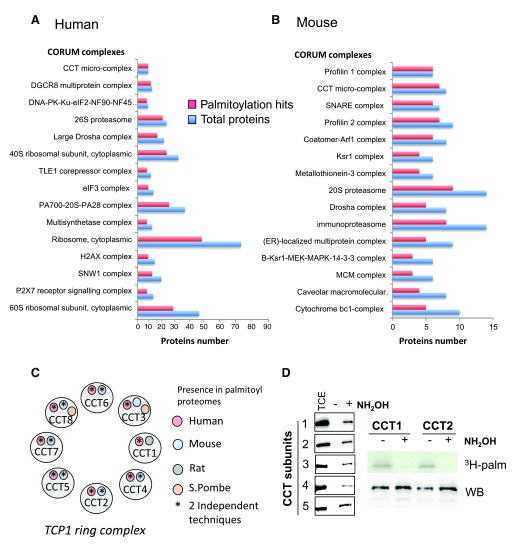
S-palmitoylation of mammalian protein complexes. **A** and **B**: Human and mouse protein complexes enriched in S-palmitoylation hits using the CORUM database. Proteins complexes containing more than 6 proteins and enriched by at least 50% of S-palmitoylation hits were selected. **C**: Representation of the TRiC complex subunits. Color circles represent the species in which each CCT subunit was identified as S-palmitoylated. The star indicated proteins identified by 2 independent techniques. **D**: Palmitoylation of the subunits was validated by Acyl-RAC on CCT1, CCT2, CCT3, CCT4 and CCT5 subunits and by ^3^H-palmitate labelling on CCT1 and CCT2 subunits. (TCE: Total cell extract, NH _2_OH: Hydroxylamine treatment, ^3^H-palm: radioactive palmitate signal, WB: Western blot signal).

***Enrichment in protein complexes.*** Intrigued by the presence of a large number of protein complexes enriched in S-palmitoylated proteins, we analysed the human and mouse palmitoyl proteome dataset using CORUM, a database of manually curated and validated mammalian protein complexes ^60^. A clear enrichment of S-palmitoylated proteins in protein complexes was observed: 18% (463/2558) of the human and 23% (217/938) of the mouse S-palmitoylation hits were part of a complex compared to the 6.8% (1455/21267) of human and 9.5% (1747/18402) of S-palmitoylation hits in their corresponding proteomes. 27 human and 20 mouse complexes possess more than 50% of their subunits S-palmitoylated ( Figure 7A and Figure 7B). Intriguingly, the majority of the components of the CCT micro complex, also called TCP-1 ring complex (TRiC), appeared to be S-palmitoylated in both human and mouse analyses ( Figure 7C). The TRiC complex is a cytosolic molecular chaperone that promotes folding of 10 to 15 percent of cellular proteins ^75^. It is composed of two identical stacked rings, each of which contains eight different subunits. We were able to experimentally confirm the S-palmitoylation of CCT components; CCT1 and CCT2 could be labeled with ^3^H-palmitate in a hydroxylamine-dependent manner ( Figure 7D). These subunits, as well as CCT3, CCT4 and CCT5, also were positive for S-palmitoylation by Acyl-RAC. CCT subunits assemble into the TRiC complex but independent roles as individual subunits proteins have also been proposed ^76^. What the functional consequence of this S-palmitoylation is remains to be established.

**Figure 8.**
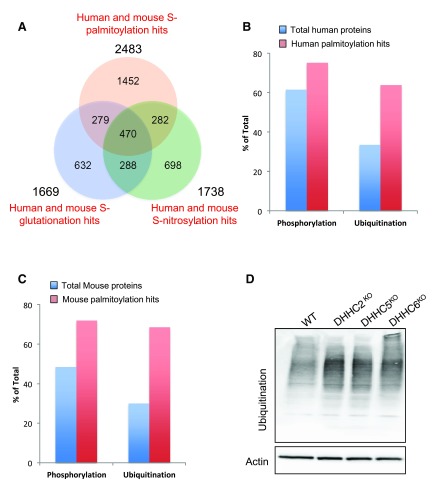
S-palmitoylation and other posttranslational modifications. **A**: Venn diagram displaying the strong co-occurrence of S-palmitoylation, S-nitrosylation and S-glutathionylation modifications within the same proteins. For this analysis human orthologs of mouse proteins were included. **B** and **C**: Percentage of phosphorylation and ubiquitination sites in human and mouse S-palmitoylation hits compared to the total protein population. **C**: Ubiquitination pattern in WT and DHHC2, 5 and 6 HAP1 cells. Total protein extracts from HAP1 cells were subject to Western blot and probe using anti-ubiquitin antibody and actin as loading control.

***PTMs and S-palmitoylation.*** S-palmitoylation is not the sole PTM that can occur on cysteine residues. They can in particular also undergo other post-translational modifications on cysteine residues such as S-nitrosylation and S-glutathionylation ^77, 78^. Site competition can thus potentially occur and has been observed ^77, 78^. To assess the potential amplitude of this competition, we compared the datasets from two available databases, Db_SNO and Db-GSH, which contain proteomic hits for S-nitrosylation and S-gluthationylation, respectively ^79, 80^. Comparison of the three datasets shows that more than 40% (1031 out of 2483) of S-palmitoylated protein hits had at least one S-nitrosylated or one S-glutathionylated site and 19% (470 proteins) had both ( Figure 8A). This strong overlap suggests that either the isolation methods share the same drawbacks or there is cross-talk between these PTMs.

Several studies, including those from our laboratory, have reported cross-talk between S-palmitoylation and phosphorylation or ubiquitination ^7, 12, 81– 88^. To investigate whether this might be a general feature, we used the Phosphosite database to analyse the presence of identified phosphorylation or ubiquitination sites among S-palmitoylation hits ^50^. We found using Uniprot annotation that 75% of the human hit proteins contained a phosphorylation site compared to 64% for total proteins. Strikingly 61% of S-palmitoylation hits contained an ubiquitination site, compare to 33% for the human proteome ( Figure 8B). Similar ratios were found for the mouse S-palmitoylated proteins ( Figure 8C). The high abundance of ubiquination sites in S-palmitoylation hits however does not imply that proteins become simultaneously S-palmitoylated and ubiquitinated. On the contrary we have found that S-palmitoylation tends to prevent ubiquitination, protecting membrane proteins for example from lysosomal or endoplasmic-reticulum associated degradation (ERAD) targeting. Consistent with our observations we found that ubiquitination was markedly increased in HAP1 cell lines knocked-out for the palmitoyltransferases ZDHHC2, ZDHHC5 or ZDHHC6 (using the CRISPR-CAS9 system) when compared to control cells ( Figure 8D).

## Conclusion

As the first database dedicated to protein S-palmitoylation, SwissPalm currently assembles 5199 proteins from 19 palmitoyl-proteomes, 7 species and 17 different tissues/cell lines. This database will be updated as new palmitoyl-proteomes appear in the literature and authors are encouraged to directly contact SwissPalm through the website ( webmaster.bbcf@epfl.ch). By curating information from multiples screens, filters could be established to improve the confidence in positive hits from palmitoyl-proteomics. In addition, SwissPalm provides various tools to ease the study of S-palmitoylation (multiple alignments of orthologous proteins and their isoforms, site prediction score from CSS-Palm and PalmPred). The combined dataset reveals that S-palmitoylation is a widespread post-translational modification that affects key biological processes. SwissPalm, by collecting and sorting information from different sources in a single web page, will accelerate research on S-palmitoylation.

## Software availability

### Software access

The code for the Ruby-on-Rails application for the SwissPalm database can be accessed online at https://github.com/bbcf/swisspalm

### Source code as at the time of publication

https://github.com/F1000Research/swisspalm.git

### Archived source code as at the time of publication

http://dx.doi.org/10.5281/zenodo.19106

### Software license

The Ruby-on-Rails application for the SwissPalm database is distributed under the MIT license.

## Supplementary materials

### Supplementary table S1

Human palmitoyl-proteome

### Supplementary table S2

Mouse palmitoyl-proteome

### Supplementary table S3

Orthologs palmitoyl-proteome

### Supplementary table S4

Topology of S-palmitoylated proteins

### Supplementary table S5

Gene Ontology analysis

### Supplementary table S6

String network analysis

## References

[1] Salaun C Greaves J Chamberlain LH: The intracellular dynamic of protein palmitoylation. *J Cell Biol.* 2010;191(7):1229–38. 10.1083/jcb.201008160 21187327 PMC3010063

[2] Blaskovic S Blanc M van der Goot FG: What does S-palmitoylation do to membrane proteins? *FEBS J.* 2013;280(12):2766–74. 10.1111/febs.12263 23551889

[3] Levental I Lingwood D Grzybek M: Palmitoylation regulates raft affinity for the majority of integral raft proteins. *Proc Natl Acad Sci U S A.* 2010;107(51):22050–4. 10.1073/pnas.1016184107 21131568 PMC3009825

[4] Abrami L Kunz B Deuquet J: Functional interactions between anthrax toxin receptors and the WNT signalling protein LRP6. *Cell Microbiol.* 2008;10(12):2509–19. 10.1111/j.1462-5822.2008.01226.x 18717822

[5] Fukata Y Fukata M: Protein palmitoylation in neuronal development and synaptic plasticity. *Nat Rev Neurosci.* 2010;11(3):161–75. 10.1038/nrn2788 20168314

[6] Lakkaraju AK Abrami L Lemmin T: Palmitoylated calnexin is a key component of the ribosome-translocon complex. *EMBO J.* 2012;31(7):1823–35. 10.1038/emboj.2012.15 22314232 PMC3321195

[7] Gauthier-Kemper A Igaev M Sündermann F: Interplay between phosphorylation and palmitoylation mediates plasma membrane targeting and sorting of GAP43. *Mol Biol Cell.* 2014;25(21):3284–99. 10.1091/mbc.E13-12-0737 25165142 PMC4214776

[8] Rossin A Durivault J Chakhtoura-Feghali T: Fas palmitoylation by the palmitoyl acyltransferase DHHC7 regulates Fas stability. *Cell Death Differ.* 2015;22(4):643–53. 10.1038/cdd.2014.153 25301068 PMC4356335

[9] Kong C Lange JJ Samovski D: Ubiquitination and degradation of the hominoid-specific oncoprotein TBC1D3 is regulated by protein palmitoylation. *Biochem Biophys Res Commun.* 2013;434(2):388–93. 10.1016/j.bbrc.2013.04.001 23578663 PMC3646999

[10] Zhu YC Li D Wang L: Palmitoylation-dependent CDKL5-PSD-95 interaction regulates synaptic targeting of CDKL5 and dendritic spine development. *Proc Natl Acad Sci U S A.* 2013;110(22):9118–23. 10.1073/pnas.1300003110 23671101 PMC3670390

[11] Linder ME Deschenes RJ: Palmitoylation: policing protein stability and traffic. *Nat Rev Mol Cell Biol.* 2007;8(1):74–84. 10.1038/nrm2084 17183362

[12] Abrami L Kunz B Iacovache I: Palmitoylation and ubiquitination regulate exit of the Wnt signaling protein LRP6 from the endoplasmic reticulum. *Proc Natl Acad Sci U S A.* 2008;105(14):5384–9. 10.1073/pnas.0710389105 18378904 PMC2291096

[13] Resh MD: Covalent lipid modifications of proteins. *Curr Biol.* 2013;23(10):R431–5. 10.1016/j.cub.2013.04.024 23701681 PMC3712495

[14] Rocks O Peyker A Kahms M: An acylation cycle regulates localization and activity of palmitoylated Ras isoforms. *Science.* 2005;307(5716):1746–52. 10.1126/science.1105654 15705808

[15] Lynch SJ Snitkin H Gumper I: The differential palmitoylation states of N-Ras and H-Ras determine their distinct Golgi subcompartment localizations. *J Cell Physiol.* 2015;230(3):610–9. 10.1002/jcp.24779 25158650 PMC4269384

[16] Greaves J Chamberlain LH: DHHC palmitoyl transferases: substrate interactions and (patho)physiology. *Trends Biochem Sci.* 2011;36(5):245–53. 10.1016/j.tibs.2011.01.003 21388813

[17] Fukata Y Iwanaga T Fukata M: Systematic screening for palmitoyl transferase activity of the DHHC protein family in mammalian cells. *Methods.* 2006;40(2):177–82. 10.1016/j.ymeth.2006.05.015 17012030

[18] El-Husseini AE Craven SE Chetkovich DM: Dual palmitoylation of PSD-95 mediates its vesiculotubular sorting, postsynaptic targeting, and ion channel clustering. *J Cell Biol.* 2000;148(1):159–72. 10.1083/jcb.148.1.159 10629226 PMC2156213

[19] Misra C Restituito S Ferreira J: Regulation of synaptic structure and function by palmitoylated AMPA receptor binding protein. *Mol Cell Neurosci.* 2010;43(4):341–52. 10.1016/j.mcn.2010.01.001 20083202 PMC3061853

[20] Hayashi T Thomas GM Huganir RL: Dual palmitoylation of NR2 subunits regulates NMDA receptor trafficking. *Neuron.* 2009;64(2):213–26. 10.1016/j.neuron.2009.08.017 19874789 PMC2788208

[21] Singaraja RR Huang K Sanders SS: Altered palmitoylation and neuropathological deficits in mice lacking HIP14. *Hum Mol Genet.* 2011;20(20):3899–909. 10.1093/hmg/ddr308 21775500 PMC3177655

[22] Liu H Abecasis GR Heath SC: Genetic variation in the 22q11 locus and susceptibility to schizophrenia. *Proc Natl Acad Sci U S A.* 2002;99(26):16859–64. 10.1073/pnas.232186099 12477929 PMC139234

[23] Mizumaru C Saito Y Ishikawa T: Suppression of APP-containing vesicle trafficking and production of beta-amyloid by AID/DHHC-12 protein. *J Neurochem.* 2009;111(5):1213–24. 10.1111/j.1471-4159.2009.06399.x 19780898

[24] Iwabuchi M Sheng H Thompson JW: Characterization of the ubiquitin-modified proteome regulated by transient forebrain ischemia. *J Cereb Blood Flow Metab.* 2014;34(3):425–32. 10.1038/jcbfm.2013.210 24301296 PMC3948117

[25] Yamamoto Y Chochi Y Matsuyama H: Gain of 5p15.33 is associated with progression of bladder cancer. *Oncology.* 2007;72(1–2):132–8. 10.1159/000111132 18025801

[26] Kang R Wan J Arstikaitis P: Neural palmitoyl-proteomics reveals dynamic synaptic palmitoylation. *Nature.* 2008;456(7224):904–9. 10.1038/nature07605 19092927 PMC2610860

[27] Oyama T Miyoshi Y Koyama K: Isolation of a novel gene on 8p21.3–22 whose expression is reduced significantly in human colorectal cancers with liver metastasis. *Genes Chromosomes Cancer.* 2000;29(1):9–15. 10.1002/1098-2264(2000)9999:9999<::AID-GCC1001>3.0.CO;2-# 10918388

[28] Birkenkamp-Demtroder K Christensen LL Olesen SH: Gene expression in colorectal cancer. *Cancer Res.* 2002;62(15):4352–63. 12154040

[29] Yeste-Velasco M Mao X Grose R: Identification of *ZDHHC14* as a novel human tumour suppressor gene. *J Pathol.* 2014;232(5):566–77. 10.1002/path.4327 24407904

[30] Blanc M Blaskovic S van der Goot FG: Palmitoylation, pathogens and their host. *Biochem Soc Trans.* 2013;41(1):84–8. 10.1042/BST20120337 23356263

[31] Roth AF Wan J Bailey AO: Global analysis of protein palmitoylation in yeast. *Cell.* 2006;125(5):1003–13. 10.1016/j.cell.2006.03.042 16751107 PMC2246083

[32] Wan J Roth AF Bailey AO: Palmitoylated proteins: purification and identification. *Nat Protoc.* 2007;2(7):1573–84. 10.1038/nprot.2007.225 17585299

[33] Forrester MT Hess DT Thompson JW: Site-specific analysis of protein S-acylation by resin-assisted capture. *J Lipid Res.* 2011;52(2):393–8. 10.1194/jlr.D011106 21044946 PMC3023561

[34] Martin BR Cravatt BF: Large-scale profiling of protein palmitoylation in mammalian cells. *Nat Methods.* 2009;6(2):135–8. 10.1038/nmeth.1293 19137006 PMC2775068

[35] Yang W Di Vizio D Kirchner M: Proteome scale characterization of human S-acylated proteins in lipid raft-enriched and non-raft membranes. *Mol Cell Proteomics.* 2010;9(1):54–70. 10.1074/mcp.M800448-MCP200 19801377 PMC2808267

[36] Dowal L Yang W Freeman MR: Proteomic analysis of palmitoylated platelet proteins. *Blood.* 2011;118(13):e62–73. 10.1182/blood-2011-05-353078 21813449 PMC3186346

[37] Ivaldi C Martin BR Kieffer-Jaquinod S: Proteomic analysis of S-acylated proteins in human B cells reveals palmitoylation of the immune regulators CD20 and CD23. *PLoS One.* 2012;7(5):e37187. 10.1371/journal.pone.0037187 22615937 PMC3355122

[38] Marin EP Derakhshan B Lam TT: Endothelial cell palmitoylproteomic identifies novel lipid-modified targets and potential substrates for protein acyl transferases. *Circ Res.* 2012;110(10):1336–44. 10.1161/CIRCRESAHA.112.269514 22496122 PMC3428238

[39] Wilson JP Raghavan AS Yang YY: Proteomic analysis of fatty-acylated proteins in mammalian cells with chemical reporters reveals *S*-acylation of histone H3 variants. *Mol Cell Proteomics.* 2011;10(3):M110.001198. 10.1074/mcp.M110.001198 21076176 PMC3047146

[40] Merrick BA Dhungana S Williams JG: Proteomic profiling of S-acylated macrophage proteins identifies a role for palmitoylation in mitochondrial targeting of phospholipid scramblase 3. *Mol Cell Proteomics.* 2011;10(10):M110.006007. 10.1074/mcp.M110.006007 21785166 PMC3205854

[41] Ren W Jhala US Du K: Proteomic analysis of protein palmitoylation in adipocytes. *Adipocyte.* 2013;2(1):17–28. 10.4161/adip.22117 23599907 PMC3627377

[42] Yount JS Moltedo B Yang YY: Palmitoylome profiling reveals S-palmitoylation-dependent antiviral activity of IFITM3. *Nat Chem Biol.* 2010;6(8):610–4. 10.1038/nchembio.405 20601941 PMC2928251

[43] Li Y Martin BR Cravatt BF: DHHC5 protein palmitoylates flotillin-2 and is rapidly degraded on induction of neuronal differentiation in cultured cells. *J Biol Chem.* 2012;287(1):523–30. 10.1074/jbc.M111.306183 22081607 PMC3249106

[44] Martin BR Wang C Adibekian A: Global profiling of dynamic protein palmitoylation. *Nat Methods.* 2011;9(1):84–9. 10.1038/nmeth.1769 22056678 PMC3248616

[45] Hemsley PA Weimar T Lilley KS: A proteomic approach identifies many novel palmitoylated proteins in Arabidopsis. *New Phytol.* 2013;197(3):805–14. 10.1111/nph.12077 23252521

[46] Wan J Savas JN Roth AF: Tracking brain palmitoylation change: predominance of glial change in a mouse model of Huntington's disease. *Chem Biol.* 2013;20(11):1421–34. 10.1016/j.chembiol.2013.09.018 24211138 PMC3880188

[47] Zhang MM Wu PY Kelly FD: Quantitative control of protein *S*-palmitoylation regulates meiotic entry in fission yeast. *PLoS Biol.* 2013;11(7):e1001597. 10.1371/journal.pbio.1001597 23843742 PMC3699447

[48] Jones ML Collins MO Goulding D: Analysis of protein palmitoylation reveals a pervasive role in *Plasmodium* development and pathogenesis. *Cell Host Microbe.* 2012;12(2):246–58. 10.1016/j.chom.2012.06.005 22901544 PMC3501726

[49] Wei X Song H Semenkovich CF: Insulin-regulated protein palmitoylation impacts endothelial cell function. *Arterioscler Thromb Vasc Biol.* 2014;34(2):346–54. 10.1161/ATVBAHA.113.302848 24357059 PMC3953448

[50] Hornbeck PV Kornhauser JM Tkachev S: PhosphoSitePlus: a comprehensive resource for investigating the structure and function of experimentally determined post-translational modifications in man and mouse. *Nucleic Acids Res.* 2012;40(Database issue):D261–70. 10.1093/nar/gkr1122 22135298 PMC3245126

[51] Altenhoff AM Schneider A Gonnet GH: OMA 2011: orthology inference among 1000 complete genomes. *Nucleic Acids Res.* 2011;39(Database issue):D289–94. 10.1093/nar/gkq1238 21113020 PMC3013747

[52] Kriventseva EV Tegenfeldt F Petty TJ: OrthoDB v8: update of the hierarchical catalog of orthologs and the underlying free software. *Nucleic Acids Res.* 2015;43(Database issue):D250–6. 10.1093/nar/gku1220 25428351 PMC4383991

[53] Kumari B Kumar R Kumar M: PalmPred: an SVM based palmitoylation prediction method using sequence profile information. *PLoS One.* 2014;9(2):e89246. 10.1371/journal.pone.0089246 24586628 PMC3929663

[54] Ren J Wen L Gao X: CSS-Palm 2.0: an updated software for palmitoylation sites prediction. *Protein Eng Des Sel.* 2008;21(11):639–44. 10.1093/protein/gzn039 18753194 PMC2569006

[55] Fresno C Fernandez EA: RDAVIDWebService: a versatile *R* interface to DAVID. *Bioinformatics.* 2013;29(21):2810–1. 10.1093/bioinformatics/btt487 23958726

[56] Huang da W Sherman BT Lempicki RA: Systematic and integrative analysis of large gene lists using DAVID bioinformatics resources. *Nat Protoc.* 2009;4(1):44–57. 10.1038/nprot.2008.211 19131956

[57] Shannon PT Grimes M Kutlu B: RCytoscape: tools for exploratory network analysis. *BMC Bioinformatics.* 2013;14:217. 10.1186/1471-2105-14-217 23837656 PMC3751905

[58] Smoot ME Ono K Ruscheinski J: Cytoscape 2.8: new features for data integration and network visualization. *Bioinformatics.* 2011;27(3):431–2. 10.1093/bioinformatics/btq675 21149340 PMC3031041

[59] Franceschini A Szklarczyk D Frankild S: STRING v9.1: protein-protein interaction networks, with increased coverage and integration. *Nucleic Acids Res.* 2013;41(Database issue):D808–15. 10.1093/nar/gks1094 23203871 PMC3531103

[60] Ruepp A Waegele B Lechner M: CORUM: the comprehensive resource of mammalian protein complexes--2009. *Nucleic Acids Res.* 2010;38(Database issue):D497–501. 10.1093/nar/gkp914 19884131 PMC2808912

[61] Vacic V Iakoucheva LM Radivojac P: Two Sample Logo: a graphical representation of the differences between two sets of sequence alignments. *Bioinformatics.* 2006;22(12):1536–7. 10.1093/bioinformatics/btl151 16632492

[62] Greaves J Gorleku OA Salaun C: Palmitoylation of the SNAP25 protein family: specificity and regulation by DHHC palmitoyl transferases. *J Biol Chem.* 2010;285(32):24629–38. 10.1074/jbc.M110.119289 20519516 PMC2915699

[63] Wurtzel JG Kumar P Goldfinger LE: Palmitoylation regulates vesicular trafficking of R-Ras to membrane ruffles and effects on ruffling and cell spreading. *Small GTPases.* 2012;3(3):139–53. 10.4161/sgtp.21084 22751447 PMC3442799

[64] Alvarez E Girones N Davis RJ: Inhibition of the receptor-mediated endocytosis of diferric transferrin is associated with the covalent modification of the transferrin receptor with palmitic acid. *J Biol Chem.* 1990;265(27):16644–55. 2398066

[65] Lynes EM Bui M Yap MC: Palmitoylated TMX and calnexin target to the mitochondria-associated membrane. *EMBO J.* 2012;31(2):457–70. 10.1038/emboj.2011.384 22045338 PMC3261551

[66] Linder ME Middleton P Hepler JR: Lipid modifications of G proteins: alpha subunits are palmitoylated. *Proc Natl Acad Sci U S A.* 1993;90(8):3675–9. 10.1073/pnas.90.8.3675 8475115 PMC46364

[67] Lu D Sun HQ Wang H: Phosphatidylinositol 4-kinase IIalpha is palmitoylated by Golgi-localized palmitoyltransferases in cholesterol-dependent manner. *J Biol Chem.* 2012;287(26):21856–65. 10.1074/jbc.M112.348094 22535966 PMC3381148

[68] Ohno Y Kashio A Ogata R: Analysis of substrate specificity of human DHHC protein acyltransferases using a yeast expression system. *Mol Biol Cell.* 2012;23(23):4543–51. 10.1091/mbc.E12-05-0336 23034182 PMC3510016

[69] Park S Patterson EE Cobb J: Palmitoylation controls the dynamics of budding-yeast heterochromatin via the telomere-binding protein Rif1. *Proc Natl Acad Sci U S A.* 2011;108(35):14572–7. 10.1073/pnas.1105262108 21844336 PMC3167557

[70] Resh MD: Fatty acylation of proteins: new insights into membrane targeting of myristoylated and palmitoylated proteins. *Biochim Biophys Acta.* 1999;1451(1):1–16. 10.1016/S0167-4889(99)00075-0 10446384

[71] Ashburner M Ball CA Blake JA: Gene ontology: tool for the unification of biology. The Gene Ontology Consortium. *Nat Genet.* 2000;25(1):25–9. 10.1038/75556 10802651 PMC3037419

[72] Caron JM Vega LR Fleming J: Single site alpha-tubulin mutation affects astral microtubules and nuclear positioning during anaphase in *Saccharomyces cerevisiae*: possible role for palmitoylation of alpha-tubulin. *Mol Biol Cell.* 2001;12(9):2672–87. 10.1091/mbc.12.9.2672 11553707 PMC59703

[73] George J Soares C Montersino A: Palmitoylation of LIM Kinase-1 ensures spine-specific actin polymerization and morphological plasticity. *Elife.* 2015;4:e06327. 10.7554/eLife.06327 25884247 PMC4429338

[74] Sha Z Brill LM Cabrera R: The eIF3 interactome reveals the translasome, a supercomplex linking protein synthesis and degradation machineries. *Mol Cell.* 2009;36(1):141–52. 10.1016/j.molcel.2009.09.026 19818717 PMC2789680

[75] Kabir MA Uddin W Narayanan A: Functional Subunits of Eukaryotic Chaperonin CCT/TRiC in Protein Folding. *J Amino Acids.* 2011;2011:843206. 10.4061/2011/843206 22312474 PMC3268035

[76] Roobol A Carden MJ: Subunits of the eukaryotic cytosolic chaperonin CCT do not always behave as components of a uniform hetero-oligomeric particle. *Eur J Cell Biol.* 1999;78(1):21–32. 10.1016/S0171-9335(99)80004-1 10082421

[77] Ho GP Selvakumar B Mukai J: S-nitrosylation and S-palmitoylation reciprocally regulate synaptic targeting of PSD-95. *Neuron.* 2011;71(1):131–41. 10.1016/j.neuron.2011.05.033 21745643 PMC3181141

[78] Hess DT Stamler JS: Regulation by *S*-nitrosylation of protein post-translational modification. *J Biol Chem.* 2012;287(7):4411–8. 10.1074/jbc.R111.285742 22147701 PMC3281651

[79] Lee TY Chen YJ Lu CT: dbSNO: a database of cysteine *S*-nitrosylation. *Bioinformatics.* 2012;28(17):2293–5. 10.1093/bioinformatics/bts436 22782549

[80] Chen YJ Lu CT Lee TY: dbGSH: a database of *S*-glutathionylation. *Bioinformatics.* 2014;30(16):2386–8. 10.1093/bioinformatics/btu301 24790154

[81] Charych EI Jiang LX Lo F: Interplay of palmitoylation and phosphorylation in the trafficking and localization of phosphodiesterase 10A: implications for the treatment of schizophrenia. *J Neurosci.* 2010;30(27):9027–37. 10.1523/JNEUROSCI.1635-10.2010 20610737 PMC6632485

[82] Tian L Jeffries O McClafferty H: Palmitoylation gates phosphorylation-dependent regulation of BK potassium channels. *Proc Natl Acad Sci U S A.* 2008;105(52):21006–11. 10.1073/pnas.0806700106 19098106 PMC2605631

[83] Dorfleutner A Ruf W: Regulation of tissue factor cytoplasmic domain phosphorylation by palmitoylation. *Blood.* 2003;102(12):3998–4005. 10.1182/blood-2003-04-1149 12920028

[84] Soskic V Nyakatura E Roos M: Correlations in palmitoylation and multiple phosphorylation of rat bradykinin B _2_ receptor in Chinese hamster ovary cells. *J Biol Chem.* 1999;274(13):8539–45. 10.1074/jbc.274.13.8539 10085087

[85] Yount JS Karssemeijer RA Hang HC: *S*-palmitoylation and ubiquitination differentially regulate interferon-induced transmembrane protein 3 (IFITM3)-mediated resistance to influenza virus. *J Biol Chem.* 2012;287(23):19631–41. 10.1074/jbc.M112.362095 22511783 PMC3365998

[86] Abrami L Leppla SH van der Goot FG: Receptor palmitoylation and ubiquitination regulate anthrax toxin endocytosis. *J Cell Biol.* 2006;172(2):309–20. 10.1083/jcb.200507067 16401723 PMC2063559

[87] Fairbank M Huang K El-Husseini A: RING finger palmitoylation of the endoplasmic reticulum Gp78 E3 ubiquitin ligase. *FEBS Lett.* 2012;586(16):2488–93. 10.1016/j.febslet.2012.06.011 22728137

[88] Valdez-Taubas J Pelham H: Swf1-dependent palmitoylation of the SNARE Tlg1 prevents its ubiquitination and degradation. *EMBO J.* 2005;24(14):2524–32. 10.1038/sj.emboj.7600724 15973437 PMC1176453

