# Supplementary material for: SwissPalm: Protein Palmitoylation database
Source: F1000Res. 2015 Jul 16;4:261. [Version 1] doi: 10.12688/f1000research.6464.1 (PMC4544385; doi:10.12688/f1000research.6464.1)
Supplement: Peer review history

# Peer review: Referee response for version 1

**DOI:** 10.5256/f1000research.6936.r9523

**Published:** 2015-08-13

**Authors:** Anthony Magee

**Affiliations:** 1 Institute of Chemical Biology, Imperial College London, London, UK

This is a very valuable contribution to the field of palmitoylation. It not only provides a wealth of data for researchers but also thoughtful interpretations.

I found the location of cysteine residues in proteins particularly helpful (Fig. 4). Also, the analysis of residues surrounding palmitoylated cysteines is informative, and in essence comes to the same conclusion that I and several others have come to (on a less systematic basis!) that there is no real "consensus" sequence for palmitoylation. This is in contrast to a few papers in the literature which erroneously purport to identify such a consensus.

The enrichment of palmitoylated proteins in complexes is interesting and suggests a possible role of fatty acyl moieties in mediating complex formation. The interplay between palmitoylation, phosphorylation, ubiquitination and other PTMs is also nicely highlighted.

The only major limitation of the paper and the website I have found is the omission of the MBOAT family of protein acyltransferases. Although only one of these is strictly probably a S-palmitoyltransferase (Hedgehog acyltransferase, Hhat), two members of the family (Porcupine and Ghrelin O-acyltransferase, GOAT) are acyltransferases that transfer fatty acyl groups onto proteins. I think it would be helpful if they could be incorporated, and would make the website more comprehensive for investigators interested in protein acylation in its broadest sense. Admittedly, Hhat is thought by some to be a **N**-palmitoyltransferase, but it is highly likely that it is a S-palmitoyltransferase and that the modified Hedgehog substrates then undergo spontaneous S-to-N acyl transfer resulting in amide-linked palmitate. Porcupine transfers the closely related fatty acyl group palmitoleate to a serine hydroxyl of Wnt proteins and GOAT transfers an octanoyl group to a serine of ghrelin, so these are not "S-palmitoyltransferases" but they share enough in common as protein fatty acyltransferases to warrant inclusion in the database. A minor point, but Hhat has also recently been identified by Konitsiotis et al. to be palmitoylated ( Konitsiotis *et al.*, 2015) so should be included in the database at the next revision.

One minor correction I would suggest - the authors generally use the term "palmitoyl proteome" throughout, which was agreed to be the appropriate term at the second dedicated meeting on protein palmitoylation in Edinburgh last year. However, the less suitable term "palmitome" has slipped through in one place, the heading at the bottom right of page 8.

I have read this submission. I believe that I have an appropriate level of expertise to confirm that it is of an acceptable scientific standard.

# Peer review: Referee response for version 1

**DOI:** 10.5256/f1000research.6936.r9527

**Published:** 2015-07-30

**Authors:** Luke Chamberlain

**Affiliations:** 1 Strathclyde Institute of Pharmacy and Biomedical Sciences, University of Strathclyde, Glasgow, UK

SwissPalm represents an extremely valuable resource for the palmitoylation research community, and the authors have undertaken a large volume of work to produce this palmitoylation database. There are also some useful and interesting comparative analyses presented in the paper. As commented by the authors, up to 10% of the proteome might be subject to palmitoylation, emphasising both the importance of this lipid modification and the importance of this new resource. Information has been curated from a growing number of protein-palmitoylome studies and from papers focused on individual palmitoylated proteins, and the database will be continuously updated.

I have only minor comments for improvement:

- Page 3, first paragraph: “while thioesterases detach it and Acyl Protein Thioesterases (APTS) which remove the acyl chain”. There appears to be some repetition here and “while thioesterases detach it” could be removed.
- Figure 1A: text states 19 palmitoyl-proteome screens were selected but first panel of Figure 1A suggests 18? Should there be 6 studies from human using chemical capture? Also text states 5199 proteins were incorporated but figure shows 5169?
- Figure 2A-C: some text is partially obscured.
- Page 8, section on “Protein information page”: the figure numbers given in text of this section do not match with the figures.
- Figure 5A: values of 1455, 2341 and 2651 are given in Figure, whereas corresponding text (pages 8 and 9) states 1453, 2339 and 2649.
- The text corresponding to Figure 7A indicates 27 human and 20 mouse protein complexes - the authors might provide some additional information as to how these values correspond with the bar graph in Figure 7A (which does not show 27/20 complexes).
- The legend of Figure 8 gives “C” rather than “D” for text corresponding to panel D.

I have read this submission. I believe that I have an appropriate level of expertise to confirm that it is of an acceptable scientific standard.

# Peer review: Referee response for version 1

**DOI:** 10.5256/f1000research.6936.r9524

**Published:** 2015-07-27

**Authors:** Howard C. Hang, Emmanuelle Thinon

**Affiliations:** 1 Laboratory of Chemical Biology and Microbial Pathogenesis, The Rockefeller University, New York, NY, USA

The database developed by Blanc and colleagues is an excellent and valuable resource for researchers working on S-palmitoylation. The database allows to visualize and compare 19 proteomics datasets published in the last few years for protein S-palmitoylation. Proteomics data often generate some false positive hits and SwissPalm-mediated improvement of confidence will help scientists interpret the results of these proteomics studies and could also use as a reference database for future proteomics studies. The other tools added to the database (multiple alignments of orthologous proteins and their isoforms, site prediction score from CSS-Palm and PalmPred) are also valuable. The database interface is well described in the paper. As a result, the database is easy to use and the data easily extracted from the website. The comparative analysis of metabolic labeling and hydroxyl amine-sensitive enrichment methods should encourage researchers to employ both methods for high-quality proteomic analysis of S-fatty-acylated proteins.

We have read this submission. We believe that we have an appropriate level of expertise to confirm that it is of an acceptable scientific standard.
